# Food Insecurity Predictors Differ for White, Multicultural, and International College Students in the United States

**DOI:** 10.3390/nu17020237

**Published:** 2025-01-10

**Authors:** Abigail A. Glick, Donna M. Winham, Mack C. Shelley

**Affiliations:** 1 Department of Food Science & Human Nutrition, Iowa State University, Ames, IA 50011, USA; aglick03@gmail.com; 2 Departments of Political Science and Statistics, Iowa State University, Ames, IA 50011, USA; mshelley@iastate.edu

**Keywords:** food security, young adults, socio ecological model, marginal food security, food frequency questionnaire, food assistance programs, African American, Hispanic, food access

## Abstract

**Background:** Higher education institutions and public health agencies in the United States (US) have recognized that food insecurity is pervasive and interferes with student learning on multiple levels. However, less research has examined food insecurity among culturally diverse college students. A cross-sectional online survey was conducted to estimate the prevalence and predictors of food insecurity for US-born White, US-born Multicultural, and International students aged 18–34 at a Midwest university. The secondary aims were to describe dietary and meal characteristics, and the use of food assistance programs, including the on-campus food pantry. **Methods:** In April 2022, 853 students completed the 10-item US Adult Food Security Module, and demographic, dietary fat intake, food attitude, food access barriers, and nutrition assistance program usage questions using a socio ecological model (SEM) framework. **Results:** Food security prevalence was 73.3% (54.7% high, 18.5% marginal) and food insecurity prevalence was 26.7% (14.4% low, 12.3% very low). Significantly more International (26.8%) and Multicultural (35.6%) students were classified as food-insecure compared to White students (19.9%; *p* < 0.001). Binomial and multinomial logistic regression models indicated that predictors of food insecurity were intrapersonal factors of race/ethnicity, poor self-reported health, being an undergraduate, and the community barriers of high food costs and limited transportation. **Conclusions:** Dietary characteristics differed more by nativity–ethnicity groups than they did by food security levels. Food cost emerged as a strong influence on food choice for the food-insecure students. International students utilized more nutrition assistance programs, including the on-campus food pantry, than other groups.

## 1. Introduction

Food security on college campuses has become an increasing concern for higher education and public health experts in the United States (US) over the past decade [1]. Defined as ‘a household-level economic and social condition of limited or uncertain access to adequate food’ [2], food insecurity affected 12.8% of household types in the U.S. in 2022 [3]. In contrast, estimates of food insecurity for students on campuses range from 20% to over 60% [1,4]. The reasons for the prevalence differences between the general population and students are complex and may be highly situation-specific [4,5,6,7,8,9].

College students may have a greater risk of food insecurity due to developing independent living skills outside of the family-of-origin household [5,6]. Grocery shopping and food preparation skills may be inadequate [7]. Lack of food outlets and restricted transportation options or reliance on public transit to grocery stores can interfere with the ability to obtain food [8]. High costs of food, little or no income, and lack of a campus meal plan can add to the burden of food access. Some aspects are related to academic performance taking precedence over employment, lack of support resources, and food safety net restrictions for students [1]. While paid employment brings monetary benefits, job hours may restrict time for grocery shopping, preparing food, and studying [9]. Most students do not qualify for federal food assistance programs like the Supplemental Nutrition Assistance Program (SNAP) [10]. Multicultural or minority students and International students may be at higher risk for food insecurity [11,12,13,14].

As emerging young adults, students may have inadequate awareness or knowledge to actively resolve their food security issues while prioritizing their academic tasks and social changes. A lack of perception of being food-insecure, or perhaps cognitive dissonance, could be reflected in reluctance to acknowledge there is a problem [15]. The effects of food insecurity go beyond hunger and worry about the next meal. Greater risks for depression, elevated stress, and poor diet were observed among students with food insecurity [13]. Social stigma may interfere with a student’s willingness to seek help. These forces can lead to poor academic performance and attrition from college [10].

Higher rates of food insecurity have been found among African American or Black students and Hispanic/Latino students [13,16,17,18,19]. Multicultural and minority students may experience additional cultural and social barriers at college. Racial discrimination and micro-aggressions toward non-White persons can negatively affect academic performance [18]. US-born Multicultural students at majority White institutions may have increased stress and health complications stemming from social bias and racism [17,18]. International students share similarities with their US-born Multicultural peers. Both groups are more likely to be food-insecure and have limited culturally sensitive social support [14,19]. Forced dietary acculturation can lead to feelings of anomie, depression, and guilt for violation of cultural food norms due to hunger and the inaccessibility of appropriate foods [20,21]. Undocumented students or those with expired visas are unlikely to seek assistance due to their vulnerability if their legal status is widely known [16]. During the early stages of the COVID-19 pandemic declaration, International students, particularly those who looked phenotypically Asian or were Muslim, regardless of citizenship, were subject to discrimination from the national administration’s policies and rhetoric [22]. The closure of university housing and visa restrictions in Spring 2020 resulted in many International students returning to their home countries. For example, at Iowa State University (ISU), between Fall 2019 and Fall 2021, Chinese national enrollment declined from 1446 to 635 [23,24]. Similar reductions occurred among Asian Indian (696 to 515) and Malaysian students (246 to 149) over the same time period [23,24].

A 2018 cross-sectional online survey at ISU, a large public Midwestern university, found that food-insecure undergraduates were more likely to be non-White, receive financial aid, live off-campus, be employed, have concerns about food costs, and report poorer-quality health. Food-insecure graduate students were predominately International, employed, lacked time to prepare food, and did not have access to cultural foods [19]. In April 2020, a second online food security survey was conducted at ISU. The timing was 4 weeks after the COVID-19 transition to online learning and the shuttering of most dormitories and campus functions. Students who were non-White, undergraduates, employed, financial aid recipients, and had lower self-efficacy for preparing meals had higher rates of food insecurity [25]. Both prior studies suggested differences in food security levels among Multicultural and International students, but had relatively small sample sizes of these subgroups.

The current research aimed to explore and compare factors associated with food security among US-born White, US-born Multicultural, and International students at ISU. The primary hypothesis was that non-White students would have a lower prevalence of food security. Binomial and more precise four-category food security levels were used in the analysis. An exploratory aim was to describe dietary and meal characteristics to determine the relationships between nativity–ethnicity and food security. A secondary objective was to assess the use of nutrition assistance resources, including a campus-based food pantry. Due to the complexity of causes and interrelationships between variables related to food insecurity, the socio ecological model (SEM) was chosen as the framework to structure the inquiry [26,27]. The synergistic levels of the SEM include intrapersonal, interpersonal, institutional, and community/policy characteristics [26].

## 2. Materials and Methods

### 2.1. Study Design and Sample Recruitment

This investigation of food security prevalence was part of an overarching research project to examine dietary acculturation, food consumption changes, and views of plant-based meat alternatives among U.S.-born White, U.S.-born Multicultural, and International students aged 18–34 years. Findings from the plant-based meat alternatives and acculturation components are reported elsewhere [28,29]. The university email addresses for students were provided by the Registrar’s Office for all students aged 18+ who permitted information release (*n* = 28,211). Separate subgroup email lists for Multicultural students (*n* = 4464) and International students (*n* = 2387), as identified by the ISU administration, were requested [30]. Survey Monkey’s online platform (www.surveymonkey.com, accessed on 7 April 2022; Momentive Inc., San Mateo, CA, USA) was used for invitations, tracking, and data collection. All International and Multicultural students received a survey invitation in early April 2022. Those with incomplete surveys and non-responders received a follow-up email one week later. A random sample of 65% of the full student email list was generated using IBM SPSS software (Version 26, IBM, Armonk, NY, USA). The random sample group received one invitation, and no reminder email. To limit confounding variations in the food environment and access to resources, only students who were taking classes on campus were eligible to participate. Students who were studying abroad, at off-campus internships, enrolled only online, or visiting from another program were excluded by survey screening questions. Surveys were reviewed for plausibility, integrity (two checks), and completeness of responses. Participants were sent a USD 5 e-gift card if they stated they wanted the incentive, gave a complete email address, and finished 75% of the survey.

### 2.2. Survey Development

Demographic questions for age, gender, marital status, children in the household, on-campus or off-campus housing, and use of a campus meal plan were drawn from the American College Health Association [31]. Personal health status was evaluated on a 5-point scale (poor = 1, excellent = 5) [32]. Information about the main food shopper [32], access to cooking facilities, and self-confidence for cooking a meal was obtained [33].

The frequency of eating homemade meals at restaurants, take-out, fast food, or free meals was measured for the previous 6 months (never, 1–3 days per month, 1–2 times per week, 3–4 days per week, almost every day) [34]. The regularity of eating the evening meal with others (1 = rarely; 5 = always) was reported [34]. Perception of diet change since starting at the university was measured (1 = strongly disagree to 5 = strongly agree) [35]. A validated 17-item dietary fat screener estimated the percentage of calories from fat [36]. Higher dietary fat intake has been associated with food insecurity in other studies [37]. The frequency options were 1 time per month or less, 2–3 times per month, 1–2 times per week, 3–4 times per week, and 5 times or more per week [36].

Food security status was determined using the United States Department of Agriculture (USDA) 10-item Adult Food Security Survey Module suitable for households without children [38,39]. The study frame of reference was the previous 6 months in order to reflect academic conditions better than the 1-year time reference utilized by the USDA. The 10-item instrument was condensed to eight questions by including the time frame element in the responses. To reduce the respondent burden of completing the whole module, all participants answered the USDA food sufficiency screener question, ‘Which statement best describes the food eaten in your household in the last 6 months?’ [38]. Students who answered ‘often not enough’, ‘sometimes not enough’, or ‘enough but not always the kinds of food wanted’ on the screening question transitioned to the food security module. Those who stated they had ‘enough of the kinds of food I want to eat’ could be considered food-secure [38]. However, as a cross-check to confirm their status, they were asked, ’How often do you run out of food before you get money again to buy more?’ [40]. Students who said they ‘never’ ran out of food were designated as food-secure [38,40]. Students who said they ‘did run out of food’ completed the food security module.

Students were asked if they had received and/or used information from ISU on how to cook meals, resources for who to talk with if they had trouble obtaining adequate food, how to apply for federal nutrition programs, and the location of food pantries and other free food source options [41]. Students reported on the frequency (1 = very often, 5 = never) at which seven food access barriers interfered with their ability to obtain adequate food, e.g., lack of transportation [41]. Inquiries were made regarding whether they, or someone they shared food expenses with, were receiving benefits from the Supplemental Nutrition Assistance Program (SNAP) and/or the Temporary Assistance for Needy Families (TANF) programs. Students answered if they had received food from any food pantry in the past 6 months. Several examples of local food pantry agencies were provided, including the ISU on-campus Students Helping Our Peers (SHOP) facility [42]. Food pantry users were asked if they knew of the SHOP and had used it. If yes, details were requested on the frequency of use, if visits were planned, and if they would be willing to provide their names and annual income to USDA so the SHOP could stock additional products. An open-ended question asked respondents what other foods or items they would like available at the SHOP.

### 2.3. Nativity and Cultural/Ethnic Classifications

Three categories of nativity, or geographic origin, and cultural/ethnic background, referred to as nativity–ethnicity in this paper, were used for analysis: US-born White, US-born Multicultural, and International students. Categorization as a Multicultural or International student originated from the designated email lists provided by the ISU Registrar and was confirmed or refined by survey responses. A 5-category generation question was adapted from the Acculturation Rating Scale for Mexican Americans [43]. Survey participants who answered 2nd through 5th generation could select up to 3 of 11 common ancestral heritages based on Iowa demographics or ‘write in’ a response, e.g., White, African American, Dutch, German, or Vietnamese [44]. They reported the language they first learned to speak from the same five choices or wrote in as above.

International students were explicitly instructed to select whether they were first-generation or born in another country. Then, they were prompted to give no more than three options to describe their cultural or ethnic background, e.g., Chinese, Iranian, Indian, etc. A list of the 12 most frequent student countries of origin based on ISU Fall 2021 enrollment data was provided along with a ‘write-in’ option [24]. Native language, or first language learned after birth, was chosen from seven options (English, Spanish, Mandarin or other Chinese dialect, Hindi, Persian (Farsi), Arabic, Korean) or ‘write-in’ [24]. International students provided the years they had spent in the US since birth. The length of time in the US included the option of ‘all of my life except for 1 year or less after birth’. The generation question had a specific option for people born in another country to US citizen parents. These questions aided the identification of several students who were US citizens born abroad, international adoptees, and immigrants who only knew the US as their home country. A few persons who emigrated to the US right after birth or prior to 9 years of age, classified themselves as in-state or out-of-state, and were not on the ISU ‘International’ email roster were grouped with the US-born Multicultural category for analysis [45,46].

### 2.4. Socio Ecological Model (SEM) Classification for Variables

The SEM framework guided the selection of the survey questions, and the interpretation of influences on food security. This holistic framework allows for a broader assessment of the interactions and dynamics between individuals and the complex layers of their environment [26]. Identifying predictive factors can guide food insecurity mitigation among diverse college student populations. Table 1 displays the variables associated with each of the four SEM levels applied in this study.

### 2.5. Data Transformations and Analysis

Online responses from Survey Monkey were downloaded in IBM SPSS format for analysis (V.26, IBM SPSS, Armonk, NY, USA). Data distributions were reviewed for normality and completeness. No data values were imputed. The food security module raw scores (ranging from 0 to 10) were computed per the USDA instructions [38]. The raw score was recoded into a binomial food-secure (0–2)/-insecure (3–10) measure, and a more precise 4-category variable (0 high, 1–2 marginal, 3–5 low, 6–10 very low) for analysis [38]. Food security studies often utilize a binomial classification only, but disaggregation between marginal, low, and very low categories may reveal more nuances with regard to the drivers of food insecurity with adequate sample sizes [39,47]. Dietary fat food frequencies were analyzed using the instrument’s online assessment tool [36,48].

In addition to classifying US-born White, US-born Multicultural, and International students, eight ethnic–cultural groups were created based on self-identification, cultural similarities, reported religion, and accommodation of small sample sizes. The final groups were (1) White (US-born Whites, Western Europeans, Canadians), (2) East Asians (Chinese, Koreans, Japanese, Malaysians), (3) Latin American (Hispanics, Latin Americans, Caribbean Islanders, Brazilians), (4) Asian Indian (including Nepalis), (5) African Americans and non-Hispanic Blacks, (6) Middle Eastern-North Africans (including Muslim Pakistanis and Bangladeshis), (7) Southeast Asians (Vietnamese, Laotians, Thais, Cambodians), and (8) Native Americans and ‘other’ respondents.

Statistical results with *p* values ≤ 0.05 were considered significant. Logistic regression models were estimated using the binomial outcome of food security or food insecurity for the whole sample and separately for the US-born White, US-born Multicultural, and International students. In addition, multinomial logistic regression (MLR), which allows for predicting the probabilities of nominal dependent variables when there are more than two groups [49], were employed to estimate differences across the four levels of food security, with high food security as the referent group. The initial models included variables from the SEM as shown in Table 1. These included binomial measures for gender, academic status, on- or off-campus housing, campus meal plans, marital status, native English speakers, US-born Multicultural status, International student status, and the eight cultural subgroups. The continuous variables of age, self-reported health status, perceived diet change, cost of food, eating meals with someone, lack of transportation, lack of cultural or ethnic foods, location of food outlets on campus, and hours of food outlets on campus were included. The final regression models were derived by sequentially removing items with significance levels greater than 0.05 unless the retention of those variables improved the model fit [49].

## 3. Results

### 3.1. Participant Response Rates and Sampling

The Multicultural response rate was 8.1% (359/4417; 35 invalid emails, 8 opt-outs, 4 duplicates), with the International response rate at 16.8% (392/2334; 17 invalid emails, 9 opt-outs, 1 duplicate). A separate random sample of survey invitations was sent to ~65% (18,337) of the 28,211 student emails. The response rate was 2.4% (444/18,099; 163 invalid emails, 43 opt-outs, 32 duplicates). A total of 1158 unique individuals started the survey. Thirty-five who were outside of the 18–34-year age range and sixty-eight who were not living near campus were excluded from advancing in the survey. Seventy-six persons failed integrity checks, and 126 cases were incomplete for variables of interest and excluded. The 853 respondents mostly lived off campus (72%), were undergraduates (64%), and were women (56%). The sample had a mean age of 22.6 ± 3.9 years. The respondents approximated the ISU campus demographics for Spring 2022 (living off campus 51%, undergraduates 83%, 45% women) [24]. In total, 37% percent of the sample were US-born Whites, 28% were US-born Multicultural students, and 35% were International students.

### 3.2. Prevalence and Classification of Food Security Levels

Overall, 49% of respondents (420/853) stated they had ‘enough of the kinds of food they want to eat’ over the past 6 months, and 51% said they did not on this screening question [38]. Those who answered ‘yes, they had enough’ were asked a cross-check question about the frequency of running out of food before the end of the month [38,40]. Of these, 77% (323/420) answered ‘never’ and were classified as food-secure based on these two screening questions [38]. Students who ran out of food (*n* = 97) and the 51% (433/853) who did not have enough of the kinds of food they wanted completed the full food security module. Supplemental Table S1 shows the individual question frequencies by nativity–ethnicity.

Based on the binomial secure/insecure classification of the food security module, 73.3% of the respondents were food-secure (Table 2). Significantly more Multicultural (35.6%) and International (26.8%) students were food-insecure compared to White students (19.9%; *p* < 0.001). Within the US-born Multicultural group, 58.3% (21/36) of African Americans and 40.4% (40/99) of Hispanics were food-insecure compared to 24.5% (13/53) of East Asian students. The binomial food secure percentages for the eight ethnic–cultural subgroups, regardless of nativity, were as follows: White 80.8%, Asian Indian 76.4%, East Asian 75.4%, Southeast Asians 73.3%, Native Americans and Other 66.7%, Hispanics 62.7%, Middle Eastern and North African 59.5%, *p* = 0.044, and African American or Blacks 50.9%, *p* < 0.001. Over 60% of the US-born White students had high food security as per the four-level classification. No nativity–ethnicity differences were observed for those with marginal food security (18.5%) or very-low-food-security students (12.3%). A significantly greater percentage of US-born Multicultural students had low food security (21.3%) compared to US-born White (10.4%) or International (13.1%) students (Table 2).

### 3.3. Demographic Characteristics and Socio Ecological Levels by Nativity–Ethnicity

Significant differences between the three nativity–ethnicity cohorts were found for age, gender, academic level, Hispanic ethnicity, English as a native language, nativity and ethnic origins, self-reported health, and food security status (all *p* < 0.005; Table 2). Most of the International cohort were graduate students, older, and non-native English speakers. Asian Indian (30.8%) and East Asian (28.4%) cultures were highly represented in the International cohort. Multicultural students were 43% Hispanic, followed by 23% East Asian and 15% African American or Black cultures. Within the Multicultural group, more women than men completed the survey. Gender representation was more balanced for White and International cohorts. Self-reported health was higher for White students compared to Multicultural and International students (Table 2).

The interpersonal and institutional variables of the SEM were significantly different according to nativity–ethnicity background (Table 3). Higher proportions of International students were married, lived off campus, and were the main food provider, compared to both US-born groups. Of the International students, 32% rarely and 21% always ate an evening meal with someone. This bimodal distribution was in significant contrast to the White and Multicultural groups. Multicultural and International groups had significantly higher institutional barriers to food access, stating they often or very often had difficulties, e.g., “no time to prepare food”. Overall, 10% of the Multicultural students did not have cooking facilities where they lived, in contrast to White (5%) and International students (4%).

Food costs, availability, and food access, including food pantry use, represented the community levels of the SEM. Responses to all of these questions were significantly different according to nativity–ethnicity. Multicultural and International students experienced issues with the cost of food and a lack of reliable transportation ‘very often’ compared to US-born White students (Table 4). Limitations on cultural foods were not a concern for White students but a frequent barrier for the other two groups. Food pantry usage was significantly higher for International and Multicultural students.

### 3.4. Dietary Fat Intake, Perceived Dietary Change, and Meal Sources

The estimated percentage of calories from fat based on the fat food frequency screener was significantly lower for International students and highest for US-born White students (*p* = 0.005; Table 5) [36,48]. The mean intake frequencies for the 17 items in the fat screener by nativity–ethnicity group are shown in Supplemental Table S2. International students had significantly lower intakes of nine items, and higher intakes of two items, compared to White and Multicultural students (all *p* ≤ 0.001).

The estimated percentage of calories from fat was not significantly different according to either the binomial or multinomial food security categories. The examination of the individual food items by the food security binomial showed higher intakes of pizza (*p* = 0.008) and whole milk (*p* = 0.010) by food-insecure students. For the multinomial variable, differences in pizza (*p* = 0.006), fried chicken (*p* = 0.016), and whole milk (*p* = 0.028) consumption were observed. Post hoc analysis indicated that food-secure students ate pizza significantly less often than the three food-insecure groups (marginal *p* = 0.017; low *p* = 0.012; very low *p* = 0.014). Food-secure students drank less whole milk than the very-low food-security group (*p* = 0.004), and ate less fried chicken than the low-food-security group (*p* = 0.003).

The majority of all nativity–ethnicity groups agreed or strongly agreed on a 5-point scale that their diet had changed since coming to the university. Significantly more of the food-insecure students agreed or strongly agreed that their diet had changed than did their food-secure peers (*p* = 0.002). For the 4-category levels, the food-secure students (3.55 ± 1.1) were significantly lower in agreeing that their diet had changed than for the marginal (3.86 ± 1.0; *p* = 0.002) and very low food security students (4.04 ± 1.0; *p* < 0.001), while the low food-secure students approached significance (3.76 ± 1.1; *p* = 0.060).

The frequency of eating homemade meals and free charitable meals was significantly higher for International students than the other two groups. Multicultural students had the highest frequencies for eating at restaurants, take-out, or campus meals, and fast food outlets, and International students the lowest. The usage of free meals was significantly different, with almost 13% of the food-insecure students eating free meals 1–3 days each month in contrast to 6% of the food-secure (*p* = 0.002). With the four-category variable, those with low and very low food security utilized free meals the most frequently. Over 92% of the food-secure and marginal students, 85% of the low-food-security group, and 81% of the very-low-food-security group never used free meals at all (*p* = 0.006).

### 3.5. Predictors of Food Insecurity in Binomial Logistic Regression Models

The initial binomial logistic regression model (food-secure/food-insecure) did an impressive job of predicting the observed variance (pseudo Nagelkerke *R*^2^ = 0.495, with 92% of the food-secure and 59.6% of the food-insecure students correctly classified) [49]. Variables with *p* values > 0.050 were sequentially removed to arrive at the most parsimonious model. The fit metrics indicate that the predictive model performed adequately (Nagelkerke pseudo-*R*^2^ = 0.494 and Cox and Snell pseudo-*R*^2^ = 0.339). The odds ratios (ORs) for students who ‘often’ felt food costs were high, non-native English speakers, who rated their health as lower, lacked reliable transportation, and were the main food provider ranged from 0.279 to 1.793. African American or Black students were over four times (OR 4.226) as likely to be food-insecure. Undergraduates were nearly four times more likely to be food-insecure (OR 3.782) than graduate students. The model was significant, and correctly classified 58.3% of food-insecure and 90.7% of food-secure students (Table 6).

Logistic regression models for White, Multicultural, and International students showed unique differences in which predictors were significant. For US-born Whites, those who were often impacted by food costs, self-reported lower quality health, lacked time to shop, and found campus food outlet hours a barrier had a higher probability of being food-insecure (OR 0.171 to 0.637). Multicultural students who did not have food cost barriers were less likely to be food-insecure (OR 0.330). However, African American–Black students were almost six times (OR 5.872) and Hispanic/Latino students nearly three times (OR 2.913) as likely to be food-insecure. Multicultural undergraduates were over four times as likely to be food-insecure than Multicultural graduate students (OR 4.410). The International students who had difficulty with food costs (OR 0.266), and lack of reliable transportation (OR 0.782) were more likely to be food insecure. Being a native English speaker (OR 6.812), African American or Black (OR 4.255), and an undergraduate (OR 3.886) increased the odds of being food-insecure. While all three subgroup models were statistically significant, the overall correct classification percentages were 91.5% for US-born White students but dropped to 77% for Multicultural and 79.9% for International students. The correct classification of those who were food-secure was best for US-born White students (96.4%), compared to 83.1% for Multicultural and 91.3% for International students. The prediction of food insecurity was best for US-born Whites (71.4%) and poorest for International students (48.8%) (Table 7, Table 8 and Table 9). The Hosmer–Lemeshow goodness-of-fit test was not significant for the four models, indicating model appropriateness [49]. However, the poor classification for the International students suggests there may be other factors associated with food insecurity not captured in the current analysis.

### 3.6. Predictors of Food Security in Multinomial Logistic Regression

Multinomial logistic regression (MLR) was used to model the four-category food security predictors. Note that food security is the reference category in the MLR, unlike in the binomial logistic regression (Table 10). The most parsimonious MLR model showed significant influences on food security with the four intrapersonal, two interpersonal, and two community–policy elements from the SEM.

Being an undergraduate student decreased the odds of food security by ~50% (odds ratio = OR 0.493; Wald *χ*^2^(1) = 18.387, *p* < 0.001). Not being African American or Black increased the odds of being food-secure substantially (OR 2.698; Wald *χ*^2^(1) = 12.4357, *p* < 0.001). A one-unit increase in self-reported health status (better health) was associated with an increase in the odds of high food security (OR 1.294; Wald *χ*^2^(1) = 8.815, *p* = 0.003). In turn, a one-unit decrease in perceived diet change (disagreed there was change) increased the odds of high food security (OR 0.736; Wald *χ*^2^(1) = 7.683, *p* = 0.021). For the two intrapersonal factors, those who were single were 41% less likely to be food-secure (OR 0.594; Wald *χ*^2^(1) = 5.484, *p* = 0.019), and for every one unit of increase in the frequency that persons ate their evening meal with someone, the odds of food security increased by almost 17% (OR 1.167; Wald *χ*^2^(1) = 7.683, *p* = 0.006. For each one-unit increase in the rarity of food costs as a barrier, the likelihood of being food-secure increased substantially (OR 3.213; Wald *χ*^2^(1) = 251.202, *p* < 0.001. Not lacking reliable transportation predicted the likelihood of high food security (OR 1.156; Wald *χ*^2^(1) = 6.321, *p* = 0.012. The deviance goodness-of-fit test indicated that the model fit the observed data well, *χ*^2^(2047) = 1356.687; *p* = 0.663 [49].

### 3.7. Awareness and Usage of Campus and Community Food Assistance Resources

A secondary goal was to gauge the use of nutrition assistance resources, including the campus-based food pantry. Students were asked about their awareness and use of food access resources at the intrapersonal, institutional, and community/policy levels (Table 11). Information on how to cook inexpensive, healthy meals was utilized most by US-born White students (15.2%), but International students had a significantly greater interest in receiving this material (53%; *p* = 0.003). International students also wanted information for resources if one needed food (32.8%), locations of food banks (33%), and how to apply to federal nutrition programs like the SNAP (37%; all *p* < 0.001). Food-insecure students did not always want information about resources.

Federal program usage was relatively low for the SNAP, TANF, WIC, and school lunches across the cohorts. Since most respondents were single without children, low utilization was expected. Multicultural students (5%) and International students (2%) were more likely to report using the SNAP. Nearly 10% of all respondents had used a food pantry in the past 6 months. Usage was more common among Multicultural and International students than US-born Whites. Of the 9.5% (*n* = 81) of respondents who said they used any food pantry, 94% were aware of the campus pantry (SHOP), 58% were International students, 57% were female, 92% lived off-campus, and 72% were single. Over 80% of food pantry users had previously used the SHOP. International students (67%) were more likely to report they had used the SHOP, and users tended to be female (57%), live off campus (92%), and single (72%). Most users waited to go to the SHOP until they ran out of food. Over 25% of Multicultural students used the campus food pantry at least once per week. About 36% of participants were willing to answer additional USDA questions to have a wider selection of foods available. International students were significantly less willing to report than other groups (*p* = 0.030) (Table 12).

Fifty-one of the sixty-six SHOP users completed an open-ended question inquiry on foods or products they would like to have available. The frequencies of their reports are shown in Supplemental Table S3. The most common responses related to ‘fruit’ (*n* = 27), vegetables (*n* = 16), and ‘produce’ (*n* = 24 responses). Protein options such as meat (*n* = 12), eggs (*n* = 12), and pulses or legumes (*n* = 8) were mentioned often. Several responses indicated a need for grains (*n* = 18), dairy (*n* = 11), fresh or perishable items (*n* = 10), and seasonings, spices, or sauces (*n* = 8). Dietary preferences that related to culture (*n* = 4), religion (*n* = 2), and allergies or intolerances (*n* = 2) were mentioned by International students and one Multicultural student but no US-born White students.

## 4. Discussion

This study examined the prevalence of food security, diet characteristics, nutrition assistance, and campus food pantry use among US-born White, US-born Multicultural, and International students attending a large public Midwestern university. The main objective of this study was to determine risk factors for food insecurity using the SEM framework, especially concerning nativity and ethnicity. Few previous studies have examined these relationships with adequate samples of non-White students, nor the application of the more precise four-category food security levels. It was hypothesized that non-White, and specifically International students, would have a higher prevalence of food insecurity based on previous campus research. The two secondary aims of examining dietary characteristics, nutrition assistance programs, and food insecurity did not have hypothesized outcomes since they were exploratory.

The food insecurity prevalence of 27% in this study was similar to that at other US universities [1,4]. The hypothesis that non-White students would be more food-insecure than Whites was supported. However, the Multicultural group (35%), including African Americans and Hispanics, had the highest food insecurity prevalence, with the International cohort second. Multicultural students were constricted by community-level SEM factors, like transportation, thus reducing food access. However, most predictors of food insecurity in the models were intrapersonal factors, which are not changeable. Other research has indicated that higher food insecurity in college among African Americans, Hispanics, and other minority groups stems from social and cultural barriers, systemic racism, and lack of safe access to social assistance programs [13,14,15,16,17,18,50,51,52,53]. Dennard et al., in a systematic review of food security research gaps with African Americans, identified that food availability is an under-evaluated barrier [51].

For the International students, the SEM interpersonal factor of being married and the positive effects of eating meals with someone else increased their likelihood of food security. Sharing meals and culture may provide interpersonal support and reduce social isolation [14,22,54]. Systemic food access difficulties were predictive of food insecurity for them as well, although as noted further below, they were the most proactive in seeking resources. Being a native English speaker was an unexpected predictor of food insecurity. Although a small portion of the International cohort, almost 50% of Black International students were native English speakers.

Further research and program design aimed at reducing current institutional, community, and policy barriers to food access faced by African American, Hispanic, and other Multicultural students are needed, particularly at predominately White institutions like ISU [52,53]. With the politicization of diversity, equity, and inclusion (DEI) programs, funding for their support in Iowa and other states has been reduced or eliminated. Hamilton et al. have detailed the benefits of DEI-type targeted programs and the necessity of similar support for successful food outreach initiatives [52].

Common predictors of food insecurity in the binomial and multinomial models across all groups were related to food access, which is directly linked to resources and purchasing power. While systemic racism can worsen food insecurity among non-White students, issues with limited income and community barriers likely affect the 20% of food-insecure US-born White students too. Food insecurity may worsen for vulnerable individuals when trying to achieve academic success, and coursework limits their ability to earn income. In turn, social stigma when accessing local food assistance resources has been given as a barrier in other studies [10,50,54]. These challenges are amplified for first-generation, non-traditional, and other students who may not have the family or peer support for pursuing a college degree.

Larger sample sizes of nativity, ethnicity, and socioeconomic status subgroups would allow for further data disaggregation to investigate the dynamic effects of the college transition, or acculturation experiences on diet and well-being [18,53,54,55,56]. While individual circumstances are complex, longitudinal studies would provide needed information on whether or not food insecurity is entrenched or transitory, and if students change in their adaptability and skills over their college years [55,57,58].

In the secondary aim of describing dietary characteristics, most items were not different according to nativity–ethnicity group or food security status. Although Multicultural students had a higher prevalence of food insecurity, their dietary fat intake was similar to that of White students. Multicultural students ate more frequently at restaurants and fast food establishments than the other two cohorts. More information on the types of foods purchased and establishments frequented by nativity–ethnicity and food security levels could indicate if these practices are time savers, and cost effective as some businesses offer discount meals. A deeper investigation of how, what, and why diets changed, and details on culinary skills, would be beneficial in relation to food security [7,10,25].

Nutrition assistance program usage was low compared to assessed need by nativity–ethnicity groups, as well as those who were food-insecure. Few younger college students are eligible for the SNAP because of eligibility requirements. Older students who are single parents or pregnant may be more likely to qualify, but meeting academic and employment stipulations plus child care can be difficult. Disclosure of nativity, or citizenship requirements, may deter students from applying for assistance if otherwise eligible [16,54].

International students were most likely to use resources and to want access to others. They may be more aware of the available food assistance resources, and more willing to utilize programs. This proactive behavior may be one reason why their food insecurity level was less than hypothesized for this study [19,25]. Another explanation may be due to the shift in International student enrollment at the university between 2018 and 2022. In 2018, there were 4009 International students [23], but only 2443 International students in the fall of 2022 [24]. Changes in the numbers and countries of origin for International students enrolled were influenced by governmental regulation changes due to the COVID-19 pandemic declaration in 2020 [22]. These changes may have altered the income level needed to study in the US. Other research studies with international students and food security have highlighted the challenges faced due to limited employment opportunities, high tuition, and financial burdens [12,20,21,22]. Further investigation is needed with International students to determine economic, political, and social impacts on selecting welcoming universities, states, and countries for study.

The information on users of the campus food pantry and polling about potential future products suggests that the food and supplies available there could better reflect the needs of the primary International student users. With ‘produce’, ‘fruit’, and protein options being the most popular items mentioned in this poll, it is clear that nutrient-dense items are requested. A request for more culturally relevant items was also frequent. However, the identified gaps in resource needs for other food-insecure students suggests there needs to be better outreach to White and Multicultural students.

Perception of need as transitory, or not recognizing that hunger or food insecurity is abnormal, may cause cognitive dissonance and stop students from seeking resources [15]. Over 30% of Multicultural students with a high prevalence of food insecurity stated they did not need information on food pantries, nor had interest in campus resources on obtaining adequate food. Qualitative research or focus groups about perceptions of food insecurity, social stigma, and biases are needed to meet student needs better [14,57,58]. Food resource information can be distributed to broader audiences so students are aware of where to receive assistance prior to having need [15]. This information is essential for future interventions at ISU, suggesting a focus on whether these individuals know the programs available to them and how to make assistance more widely available. Focusing on institutional enablers such as increasing scholarships and funding, community factors like improving bus access and public transportation, and policy changes like advocating for college student eligibility in public programs such as SNAP could benefit all students.

## 5. Limitations

Despite the strengths of this study, there are several limitations. All variables were self-reported. Stigma associated with the topic of food insecurity may have introduced inaccuracies or social desirability in responses. While the US-born White sample was randomly selected, the Multicultural and International samples were not. The response rate for the latter two groups was less than 20%, suggesting that results for those groups may be biased. Other variables not measured in this study that may influence food insecurity include employment, being a first-generation college student, stigma about utilizing food assistance programs, receiving financial aid, and an estimate of income [11,58]. There are limitations to the food security assessment tools that may result in misclassification of individuals or not detecting other influential factors. The nativity–ethnicity groupings are imperfect in their structure. The analysis may have missed the nuances of food security issues by combining small numbers of diverse students due to respondent privacy reasons. Larger sample sizes of the smaller subgroups might allow for further disaggregation and make it easier to identify the relevant impact of acculturation or transition experiences [18,59]. The study results apply only to the data analytic sample and are not generalizable to the university, other populations, or other settings.

## 6. Conclusions

Nativity–ethnicity variables are inseparable from food security and require integrative modeling like the SEM or other systems thinking models to evaluate the nuances. The results support data collection on subgroup populations for progress on improving food security for all students. The four-level food security classifications should be used routinely and should be combined only after an examination indicates insufficient sample size to support meaningful analysis. This study utilized the SEM adjusted for collegiate populations, for which most of the significant associations were located in the intrapersonal level of the framework. For the most part, intrapersonal characteristics are immutable. Thus, change is needed at the SEM interpersonal, institutional, community, and policy levels to reduce systemic biases in food availability. Public health messaging and programming should focus on promoting knowledge of systemic barriers at the interpersonal, institutional, and community levels to avoid the stigmatization of populations based on intrapersonal characteristics. Training of public health professionals, dietitians, health coaches, and college counselors should incorporate education in this sector to give culturally and financially appropriate recommendations [59].

## Figures and Tables

**Table 1:** Influences on food security status within the socio ecological model (SEM).

Intrapersonal:	Age, gender, academic level, native English speaker, nativity–ethnicity, self-reported health status, food security status, cooking self-efficacy, diet change at university
Interpersonal:	Marital status, children in household, housing location, eats evening meal with another, main food provider
Institutional:	Campus meal plan, cooking facilities, no time to prepare food, no time to grocery shop, campus food outlet hours, location of campus food outlets, distribution of resources, use of campus food pantry
Community/Policy:	Food costs, availability of cultural foods, lack of transportation, use of any food pantry

**Table 2:** Socio-ecological model intrapersonal characteristics by nativity–ethnicity background among Midwest university students (%; *n*).

	Total (*n* = 853)	US White (37%; 316)	Multicultural (28%; 239)	International (35%; 298)	*p*
Age in years ($\bar{x}$ ± SD)	22.6 ± 3.9	21.2 ± 2.6	20.9 ± 2.9	25.6 ± 4.2	<0.001
INTRAPERSONAL	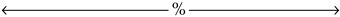				
Food Security—Binomial					<0.001
Secure	73.3	80.1 _a_	64.4 _b_	73.2 _c_	
Insecure	26.7	19.9 _a_	35.6 _a_	26.8 _c_	
Food Security—4 Categories					0.002
High	54.7	61.4 _a_	48.5 _b_	52.7 _b_	
Marginal	18.5	18.7 _a_	15.9 _a_	20.5 _a_	
Low	14.4	10.4 _a_	21.3 _b_	13.1 _a_	
Very Low	12.3	9.5 _a_	14.2 _a_	13.8 _a_	
Gender					0.006
Male	43.7	42.1 _a_	37.2 _a_	50.7 _b_	
Female	56.3	57.9 _a_	62.8 _a_	49.3 _b_	
Academic level					<0.001
Undergraduate	63.9	83.9 _a_	83.7 _a_	26.8 _b_	
Graduate	36.1	16.1 _a_	16.3 _a_	73.2 _b_	
Hispanic					<0.001
No	84.5	100 _a_	56.7 _b_	90.3 _c_	
Yes	15.5	0 _a_	43.3 _b_	9.7 _c_	
Native English speaker					<0.001
No	37.7	0 _a_	23.0 _b_	89.6 _c_	
Yes	62.3	100 _a_	77.0 _b_	10.4 _c_	
Ancestral Origin					<0.001
White—European	40.2	100	0.4	8.7	
East Asian	16.2	0	22.2	28.4	
Latin America	14.8	0	41.4	9.0	
Asian Indian	12.4	0	5.9	30.8	
African American—Black	6.4	0	15.1	6.4	
Middle Eastern	4.3	0	0	12.4	
Southeast Asian	3.5	0	7.5	4.0	
Native American—Other	2.1	0	7.5	0	
Self-reported health					0.005
Poor–Fair	15.4	13.0 _a_	15.9 _a_	17.4 _a_	
Good	41.4	36.1 _a_	46.0 _b_	43.3 _b_	
Very Good	34.3	41.5 _a_	32.6 _b_	28.2 _b_	
Excellent	8.9	9.5 _a,b_	5.4 _b_	11.1 _a_	

Each subscript letter (a, b, c) denotes a subset of categories whose column percentages do not differ significantly from each other at the 0.05 level.

**Table 3:** Socio ecological model interpersonal and institutional variables by nativity–ethnicity among Midwest university students (%; *n*).

	Total (*n* = 853)	US White (37%; 316)	Multicultural (28%; 239)	International (35%; 298)	*p*
INTERPERSONAL	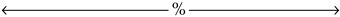				
Marital Status					<0.001
Single	83.9	88.3 _a_	87.4 _a_	76.5 _b_	
Married/Cohabitating	16.1	11.7 _a_	12.6 _a_	26.5 _b_	
Children < 18 years					<0.001
No children	97.3	99.4 _a_	98.3 _a_	94.3 _b_	
Has children	2.7	0.6 _a_	1.7 _a_	5.7 _b_	
Housing location					<0.001
On campus or parents	28.3	32.9 _a_	39.7 _a_	14.1 _b_	
Off campus	71.7	67.1 _a_	60.3 _a_	85.9 _b_	
Eats evening meal with someone					<0.001
Rarely (0–10%)	26.0	21.8 _a_	23.4 _a_	32.6 _b_	
Seldom (25%)	19.5	19.9 _a_	20.5 _a_	18.1 _a_	
Sometimes (50%)	15.6	16.8 _a_	19.7 _a_	11.1 _b_	
Most times (75%)	23.6	27.8 _a_	25.1 _a_	17.8 _b_	
Always (100%)	15.4	13.6 _a_	11.3 _a_	20.5 _b_	
Main food provider status					0.006
Someone else	40.1	41.8 _a_	46.4 _a_	33.2 _b_	
Main food provider	59.9	58.2 _a_	53.6 _a_	66.8 _b_	
INSTITUTIONAL					
Campus meal plan					<0.001
No campus meal plan	69.1	64.9 _a_	55.2 _a_	84.8 _b_	
Has campus meal plan	30.9	35.1 _a_	44.8 _a_	15.2 _b_	
Has cooking facilities					0.010
No	6.1	5.1 _a_	10.0 _b_	4.0 _a_	
Yes	93.9	94.9 _a_	90.0 _b_	96.0 _a_	
No time to prepare food					0.010
Very Often–Often	42.6	35.4 _a_	44.0 _b_	48.9 _b_	
Sometimes	31.6	33.8 _a_	32.3 _a_	28.9 _a_	
Rarely–Never	25.8	30.9 _a_	23.8 _a,b_	22.2 _b_	
No time to grocery shop					0.039
Very Often–Often	32.5	26.5 _a_	36.3 _b_	35.4 _b_	
Sometimes	28.0	27.8 _a_	27.4 _a_	28.7 _a_	
Rarely–Never	39.5	45.7 _a_	36.3 _b_	36.0 _b_	
Locations of campus food outlets					<0.001
Very Often–Often	18.0	11.2 _a_	20.3 _b_	22.9 _b_	
Sometimes	18.5	12.5 _a_	21.5 _b_	22.2 _b_	
Rarely–Never	63.5	76.4 _a_	58.1 _b_	54.9 _b_	
Hours of campus food outlets					0.002
Very Often–Often	18.6	13.1 _a_	25.8 _b_	18.4 _a_	
Sometimes	24.3	23.2 _a_	23.4 _a_	25.9 _a_	
Rarely–Never	57.2	63.7 _a_	50.8 _b_	55.7 _b_	

Each subscript letter (a, b) denotes a subset of categories whose column percentages do not differ significantly from each other at the 0.05 level.

**Table 4:** Socio ecological model community variables by nativity–ethnicity for Midwest university students (%; *n*).

	Total (*n* = 853)	US White (37%; 316)	Multicultural (28%; 239)	International (35%; 298)	*p*
COMMUNITY	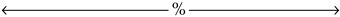				
Cost of food					<0.001
Very Often–Often	26.2	19.2 _a_	28.2 _b_	31.6 _b_	
Sometimes	28.3	25.2 _a_	30.2 _a_	29.7 _a_	
Rarely–Never	45.5	55.6 _a_	41.5 _b_	38.6 _b_	
Cultural foods not available					<0.001
Very Often–Often	28.7	4.8 _a_	33.9 _b_	48.3 _c_	
Sometimes	16.7	5.1 _a_	22.2 _b_	23.8 _b_	
Rarely–Never	54.7	90.1 _a_	44.0 _b_	27.9 _c_	
Lack of reliable transportation					<0.001
Very Often–Often	14.7	5.1 _a_	14.6 _b_	24.4 _c_	
Sometimes	16.5	11.2 _a_	19.4 _b_	19.4 _b_	
Rarely–Never	68.8	83.7 _a_	66.0 _b_	56.2 _c_	
Used any food pantry					<0.001
Yes	90.5	96.5 _a_	90.0 _b_	84.6 _b_	
No	9.5	3.5 _a_	10.0 _b_	15.4 _b_	

Each subscript letter (a, b, c) denotes a subset of categories whose column percentages do not differ significantly from each other at the 0.05 level.

**Table 5:** Dietary intakes, meal sources, and food choice influences by nativity–ethnicity among Midwest university students (%; *n*).

	Total (*n* = 853)	US White (37%; 316)	Multicultural (28%; 239)	International (35%; 298)	*p*
	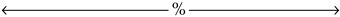				
Percent calories from fat					0.005
Less than 30%	10.8	8.2 _a_	8.8 _a_	15.1 _b_	
30–35% average	30.6	27.2 _a_	29.7 _a,b_	34.9 _b_	
36–40% high	35.2	37.0 _a_	36.8 _a_	31.9 _a_	
40–50% very high	23.4	27.5 _a_	24.7 _a,b_	18.1 _b_	
Diet has changed at the university					0.046
Strongly disagree–Disagree	15.7	17.7 _a_	14.2 _a_	14.8 _a_	
Neutral	16.0	11.4 _a_	16.7 _a,b_	20.2 _b_	
Agree–Strongly agree	68.3	70.9 _a_	69.0 _a_	65.0 _a_	
Meal location frequency ^1^	$\bar{x}$ ± SD				
Homemade food or meals	3.83 ± 1.3	3.73 ± 1.3 _a_	3.59 ± 1.3 _a_	4.12 ± 1.1 _b_	<0.001
Restaurant, take-out, or campus	3.03 ± 1.2	3.05 ± 1.2 _a_	3.32 ± 1.2 _b_	2.79 ± 1.0 _c_	<0.001
Fast food, e.g., McDonalds	2.21 ± 0.8	2.18 ± 0.8 _a_	2.39 ± 0.9 _b_	2.09 ± 0.8 _a_	<0.001
Free meals from charitable groups	1.13 ± 0.4	1.08 ± 0.4 _a_	1.12 ± 0.4 _a_	1.20 ± 0.5 _b_	0.007

Each subscript letter (a, b, c) denotes a subset of categories whose column percentages do not differ significantly from each other at the 0.05 level;. ^1^ Meal location values: 1 = never, 2 = 1–3 days per month, 3 = 1–2 times per week, 4 = 3–4 days per week, 5 = almost every day [33].

**Table 6:** Logistic regression model of food insecurity of Midwest university students (*n* = 853).

			95% Confidence Interval		
	B (SE)	Sig.	Lower	Odds Ratio	Upper
Cost of food as barrier	−1.276 (0.104)	<0.001	0.227	0.279	0.342
Native English speaker (1)	−1.208 (0.322)	<0.001	0.159	0.299	0.561
African American/Black	1.441 (0.378)	<0.001	2.014	4.226	8.867
Graduate–Undergraduate (1)	1.330 (0.288)	<0.001	2.150	3.782	6.656
Self-reported health	−0.309 (0.117)	0.008	0.584	0.734	0.923
Main food provider	0.584 (0.220)	0.008	1.164	1.793	2.761
Not an International student	−0.791 (0.354)	0.025	0.226	0.453	0.907
Lack of reliable transportation	−0.161 (0.079)	0.042	0.730	0.852	0.994
Constant	3.991 (0.685)	<0.001		54.116	
Percent correct	Food Secure	90.7		Overall	82.1
	Food Insecure	58.3			
Model significance	<0.001				

**Table 7:** Logistic regression model of food insecurity of US-born White students (*n* = 316).

			95% Confidence Interval		
	B (SE)	Sig.	Lower	Odds Ratio	Upper
Cost of food as barrier	−1.766 (0.249)	<0.001	0.105	0.171	0.279
Self-reported health	−0.947 (0.245)	<0.001	0.240	0.388	0.627
No time to shop	−0.493 (0.189)	0.009	0.421	0.611	0.885
Campus food outlet locations	0.459 (0.203)	0.024	1.064	1.583	2.355
Hours of campus food outlets	−0.451 (0.204)	0.027	0.427	0.637	0.951
Constant	8.380 (1.389)	<0.001		4357.113	
Percent correct	Food-Secure	96.4		Overall	91.4
	Food-Insecure	71.4			
Model significance	<0.001				

**Table 8:** Logistic regression model of food insecurity of US-born Multicultural students (*n* = 239).

			95% Confidence Interval		
	B (SE)	Sig.	Lower	Odds Ratio	Upper
Cost of food as barrier	−1.109 (0.162)	<0.001	0.240	0.330	0.453
African American/Black	1.770 (0.519)	0.001	2.123	5.872	16.241
Graduate–Undergraduate (1)	1.484 (0.541)	0.006	1.526	4.410	12.741
Hispanic/Latino	1.069 (0.388)	0.006	1.363	2.913	6.225
Main food provider	0.673 (0.363)	0.006	0.962	1.960	3.992
Constant	0.300 (0.769)	0.697		1.350	
Percent correct	Food-Secure	83.1		Overall	77.0
	Food-Insecure	65.9			
Model significance	<0.001				

**Table 9:** Logistic regression model of food insecurity of International students (*n* = 298).

			95% Confidence Interval		
	B (SE)	Sig.	Lower	Odds Ratio	Upper
Cost of food as barrier	−1.325 (0.183)	<0.001	0.186	0.266	0.380
Graduate–Undergraduate (1)	1.357 (0.386)	<0.001	1.824	3.886	8.276
Native English speaker	1.919 (0.687)	0.005	1.772	6.812	26.182
Lack of reliable transportation	−0.246 (0.118	0.037	0.620	0.782	0.985
African American/Black	1.448 (0.732)	0.048	1.014	4.255	17.863
Constant	1.142 (0.816)	0.162		3.132	
Percent correct	Food-Secure	91.3		Overall	79.9
	Food-Insecure	48.8			
Model significance	<0.001				

**Table 10:** Multinomial logistic regression model for predictors of food security level among US-born White, US-born Multicultural, and International college students.

			95% Confidence Interval for Odds Ratio		
	B (SE)	Sig.	Lower	Odds Ratio	Upper
LEVELS OF FOOD SECURITY					
Very low food security	2.604 (0.5449)	<0.001	4.646	13.516	39.322
Low food security	4.019 (0.5543)	<0.001	18.775	55.637	164.870
Marginal food security	5.311 (0.5683)	<0.001	66.505	202.596	617.173
INTRAPERSONAL					
Undergraduate (0)–Graduate (1)	−0.707 (0.1649)	<0.001	0.357	0.493	0.681
Not African American or Black	0.953 (0.2838)	0.001	1.554	2.698	4.683
Self-reported health status	0.258 (0.0867)	0.003	1.091	1.294	1.533
Diet has changed at college	−0.166 (0.0718)	0.021	0.736	0.847	0.975
INTERPERSONAL					
Marital status	−0.521 (0.2224)	0.019	0.384	0.594	0.919
Eats evening meal with someone	0.155 (0.0558)	0.006	1.046	1.167	1.302
COMMUNITY–POLICY					
Cost of food	1.167 (0.0736)	<0.001	2.780	3.212	3.711
Lack of reliable transportation	0.145 (0.0578)	0.012	1.033	1.156	1.295

**Table 11:** Resource and food assistance program usage by nativity–ethnicity among Midwest university students (%; *n*).

Resource and Program Usage	Total (*n* = 853)	US White (37%; 316)	Multicultural (28%; 239)	International (35%; 298)	*p*
INTRAPERSONAL					
How to cook simple, low-cost, healthy meals					
Received and used	11.6	15.2 _a_	8.8 _a_	10.1 _a_	0.003
Received but did not need	15.2	15.2 _a_	17.6 _a_	13.2 _a_	
Not received but would like	44.9	38.9 _a_	41.8 _a_	53.7 _b_	
Not received and do not need	28.3	30.7 _a_	31.8 _a_	23.0 _a_	
INSTITUTIONAL					
Campus resources if having trouble obtaining enough food					
Received and used	6.0	3.8 _a_	8.8 _b_	6.1 _a,b_	<0.001
Received but did not need	28.7	33.9 _a_	31.9 _a_	20.6 _b_	
Not received but would like	21.1	6.3 _a_	18.1 _b_	39.2 _c_	
Not received and did not need	44.2	56.0 _a_	41.2 _b_	34.1 _b_	
COMMUNITY					
Location of food pantries, food banks, or free food sources					
Received and used	15.8	5.7 _a_	15.1 _b_	27.2 _c_	<0.001
Received but did not need	34.0	42.4 _a_	39.5 _a_	20.8 _b_	
Not received but would like	18.7	8.2 _a_	14.7 _b_	32.9 _c_	
Not received and did not need	31.5	43.7 _a_	30.7 _b_	19.1 _c_	
POLICY					
How to apply for federal food assistance programs (e.g., SNAP ^1^)					
Received and used	4.3	3.2 _a_	6.3 _a_	4.1 _a_	<0.001
Received but did not need	13.7	17.1 _a_	15.9 _a_	8.4 _b_	
Not received but would like	21.6	7.9 _a_	20.9 _b_	36.8 _c_	
Not received and did not need	60.3	71.8 _a_	56.9 _b_	50.7 _b_	

^1^ Supplemental Nutrition Assistance Program; same subscript letters (a, b, etc.) indicate column proportions that are not significantly different from each other; *p* ≤ 0.05 are significant.

**Table 12:** Usage of food assistance programs and on-campus food pantry by nativity–ethnicity among Midwest university students (%; *n*).

	Total (*n* = 853)	US White (37%; 316)	Multicultural (28%; 239)	International (35%; 298)	*p*
Food assistance program usage	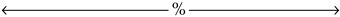				
Uses SNAP _1_	2.5	0.9 _a_	5.0 _b_	2.0 _a,b_	0.008
Uses TANF _2_	0.9	0.6	0.8	1.3	n.s.
Free–reduced school meals	1.5	0.6	0	0.6	n.s.
WIC _3_	1.2	0.3 _a_	0.8 _a_	2.3 _b_	0.055
Uses any food pantry	9.5	3.5 _a_	10.0 _b_	15.4 _b_	<0.001
Of the 9.5% (81/853) who said they used any food pantry…					
Used campus food pantry (SHOP)					<0.001
Yes, in past 6 months	77.8	45.5 _a_	70.8 _a_	89.1 _b_	
Yes, but not in past 6 months	3.7	0 _a_	4.2 _a_	4.3 _a_	
No, but know about it	12.3	54.5 _a_	12.5 _b_	2.2 _b_	
No, and do not know about it	6.2	0 _a_	12.5 _a_	4.3 _a_	
Of the 80% (63/81) who said they used the campus food pantry…					
When do you use the SHOP?					n.s.
Wait until I run out of food	76.9	100.0	66.7	78.6	
Regularly to help with budget	23.1	0	33.3	21.4	
Frequency of using the SHOP					n.s.
Multiple times per week	3.0	0	5.6	2.3	
Once a week	9.1	0	22.2	4.7	
1–3 times a month	39.4	40.0	27.8	44.2	
Less than once per month	48.5	60.0	44.4	48.8	
Willing to answer USDA questions to allow for more food items?					0.030
Yes, would answer	36.4	60.0 _a_	50.0 _a_	27.9 _a_	
No, do not want to answer	28.8	0 _a,b_	5.6 _b_	41.9 _a_	
Maybe, need more information	34.8	40.0 _a_	44.4 _a_	30.2 _a_	
Are there other foods you want at the SHOP?					n.s.
Yes	75.8	100.0	77.8	72.1	
No	24.2	0	22.2	27.9	

_1_ SNAP = Supplemental Nutrition Assistance Program; _2_ TANF = Temporary Aid for Needy Families. _3_ WIC = Special Supplemental Nutrition Program for Women, Infants, and Children. Same subscript letters (a, b) indicate column proportions that are not significantly different from each other; *p* ≤ 0.05 are significant; n.s. = non-significant.

## Data Availability

The data are available upon request from the corresponding author due to safeguarding the privacy of disaggregated groups.

## Supplementary Materials

The following supporting information can be downloaded at: https://www.mdpi.com/article/10.3390/nu17020237/s1, Table S1: Food security category classification and individual module responses for the past 6 months by nativity–ethnicity among Midwest university students [3,38,40]; Table S2: Mean consumption frequency of individual food items from the dietary fat food frequency screener nativity–ethnicity groups of Midwest university students [36]; Table S3: Requested food items from on-campus food pantry users by nativity–ethnicity from Midwest university students.

## References

[1] Goldman B.J., Freiria C.N., Landry M.J., Arikawa A.Y., Wright L. Research trends and gaps concerning food insecurity in college students in the United States: A scoping review.

[2] U.S. Department of Agriculture Economic Research Service Definitions of Food Security. Published 25 October 2023. https://www.ers.usda.gov/topics/food-nutrition-assistance/food-security-in-the-u-s/definitions-of-food-security/#:~:text=Food%20insecurity%E2%80%94the%20condition%20assessed.

[3] Rabbitt M.P., Hales L.J., Burke M.P., Coleman-Jensen A. (2023). Household Food Security in the United States in 2022 (Report No. ERR-325).

[4] Adamovic E., Newton P., House V. (2022). Food insecurity on a college campus: Prevalence, determinants, and solutions. J. Am. Coll. Health.

[5] Hagedorn R.L., Olfert M.D. (2018). Food insecurity and behavioral characteristics for academic success in young adults attending an Appalachian university. Nutrients.

[6] Zigmont V., Linsmeier A.M. (2019). Understanding the why of college student food insecurity. J. Hunger. Environ. Nutr..

[7] Knol L.L., Robb C.A., McKinley E.M., Wood M. (2019). Very low food security status is related to lower cooking self-efficacy and less frequent food preparation behaviors among college students. J. Nutr. Educ. Behav..

[8] Kim Y., Murphy J., Craft K., Waters L., Gooden B.I. (2024). “It’s just a constant concern in the back of my mind”: Lived experiences of college food insecurity. J. Am. Coll. Health.

[9] Fortin K., Harvey S., White S.S. (2021). Hidden Hunger: Understanding the complexity of food insecurity among college students. J. Am. Coll. Nutr..

[10] Richards R., Stokes N., Banna J., Cluskey M., Bergen M., Thomas V., Bushnell M., Christensen R. (2023). A comparison of experiences with factors related to food insecurity between food secure and food insecure college students: A qualitative study. J. Acad. Nutr. Diet..

[11] Payne-Sturges D.C., Tjaden A., Caldeira K.M., Vincent K.B., Arria A.M. (2020). Student hunger on campus: Food insecurity among college students and implications for academic institutions. Am. J. Health Promot..

[12] Soldavini J., Andrew H., Berner M. (2022). Campus-based food insecurity: The case of international students at a Southeastern University. J. Stud. Aff. Res. Pr..

[13] Reeder N., Tapanee P., Persell A., Tolar-Peterson T. (2020). Food insecurity, depression, and race: Correlations observed among college students at a university in the southeastern United States. Int. J. Environ. Res. Public Health.

[14] Shi Y., Lukomskyj N., Allman-Farinelli M. (2021). Food access, dietary acculturation, and food insecurity among International tertiary education students: A scoping review. Nutrition.

[15] Ong A.S.-J., Frewer L., Chan M.-Y. (2017). Cognitive dissonance in food and nutrition—A review. Crit. Rev. Food Sci. Nutr..

[16] Valadez M., Ayón C., Enriquez L.E., Jefferies J. (2021). Legal vulnerability and campus environment: Assessing factors that affect the academic engagement of undocumented college students. J. Latinos Educ..

[17] Bowen S., Elliott S., Hardison-Moody A. (2021). The structural roots of food insecurity: How racism is a fundamental cause of food insecurity. Sociol. Compass.

[18] Stevens C., Liu C.H., Chen J.A. (2018). Racial/ethnic disparities in US college students’ experience: Discrimination as an impediment to academic performance. J. Am. Coll. Health.

[19] Hiller M.B., Winham D.M., Knoblauch S.T., Shelley M.C. (2021). Food security characteristics vary for undergraduate and graduate students at a Midwest University. Int. J. Environ. Res. Public Health.

[20] Alakaam A.A., Castellanos D.C., Bodzio J., Harrison L. (2015). The factors that influence dietary habits among International students in the United States. J. Int. Stud..

[21] Alakaam A., Willyard A. (2020). Eating habits and dietary acculturation effects among international college students in the United States. AIMS Public Heal.

[22] Maleku A., Kim Y.K., Kirsch J., Um M.Y., Haran H., Yu M., Moon S.S. (2022). The hidden minority: Discrimination and mental health among international students in the US during the COVID-19 pandemic. Heal Soc. Care Community.

[23] Iowa State University (2019). Fall Semester 2019 Enrollment Undergraduates. https://www.registrar.iastate.edu/sites/default/files/uploads/stats/gender/LRSPC%20-%20F19.pdf.

[24] Iowa State University (2023). Enrollment by Race and Ethnicity. Fact Book: Enrollment..

[25] Davitt E.D., Heer M.M., Winham D.M., Knoblauch S.T., Shelley M.C. (2021). Effects of COVID-19 on university student food security. Nutrients.

[26] Bronfenbrenner U. (1994). Ecological Models of Human Development.

[27] Hayden J. (2009). Introduction to Health Behavior Theory.

[28] Luong R.H., Winham D.M., Shelley M.C., Glick A.A. (2024). Plant-Based Meat Alternatives Predicted by Theory of Planned Behavior Among Midwest Undergraduates. Foods.

[29] Luong R.H. (2022). Plant-Based Meat Alternatives Consumption and Acculturation among International College Students. Master’s Thesis.

[30] Iowa State University Office of Multicultural Student Affairs. https://multicultural.dso.iastate.edu/.

[31] American College Health Association (2022). National College Health Assessment. American College Health Association—National College Health Assessment. https://www.acha.org/wp-content/uploads/2024/07/NCHA-III_SPRING_2022_UNDERGRAD_REFERENCE_GROUP_EXECUTIVE_SUMMARY.pdf.

[32] Lando A., Verrill L., Wu F. (2021). FSANS FDA’s Food Safety and Nutrition Survey 2019 Survey. https://www.fda.gov/media/146532/download?attachment.

[33] Richards R., Brown L.B., Williams D.P., Eggett D.L. (2017). Developing a questionnaire to evaluate college students’ knowledge, attitude, behavior, self-efficacy, and environmental factors related to canned foods. J. Nutr. Educ. Behav..

[34] Green S.H., Glanz K. (2015). Development of the perceived nutrition environment measures survey. Am. J. Prev. Med..

[35] Stephenson M. (2000). Development and validation of the Stephenson Multigroup Acculturation Scale (SMAS). Psychol. Assess..

[36] Block G., Gillespie C., Rosenbaum E.H., Jenson C. (2000). A rapid food screener to assess fat and fruit and vegetable intake. Am. J. Prev. Med..

[37] Mello J.A., Gans K.M., Risica P.M., Kirtania U., Strolla L.O., Fournier L. (2010). How is food insecurity associated with dietary behaviors? An analysis with low-income, ethnically diverse participants in a nutrition intervention study. J. Am. Diet. Assoc..

[38] Economic Research Service (2012). U.S. Adult Food Security Survey Module: Three Stage Design, with Screeners.

[39] Nikolaus C.J., Ellison B., Nickols-Richardson S.M. (2019). Are estimates of food insecurity among college students accurate? Comparison of assessment protocols. PLoS ONE.

[40] Winham D.M., Palmer S.M., Florian T.L.A., Shelley M.C. (2018). Health behaviors among low-income Hispanic and non-Hispanic White women. Am. J. Heal Behav..

[41] Global Food Initiative: Food and Housing Security at the University of California. University of California: Global Food Initiative. https://www.ucop.edu/global-food-initiative/_files/food-housing-security.pdf.

[42] Iowa State University Food Security Support. Campus Food Pantry (SHOP). https://cyclonehealth.iastate.edu/basic-needs-support.

[43] Cuellar I., Arnold B., Maldonado R. (1995). Acculturation rating scale for Mexican Americans-II: A revision of the original ARSMA scale. Hisp. J. Behav. Sci..

[44] Iowa State Data Center Race & Ethnicity. https://www.iowadatacenter.org/index.php/data-by-subject/race-ethnicity.

[45] Blanchet R., Sanou D., Batal M., Nana C.P., Giroux I. (2017). Draw and tell: Dietary acculturation as lived by Black immigrant children of African and Caribbean descent residing in Canada. J. Nutr. Educ. Behav..

[46] Mulasi-Pokhriyal U., Smith C., Franzen-Castle L. (2011). Investigating dietary acculturation and intake among US-born and Thailand/Laos-born Hmong-American children aged 9–18 years. Public Health Nutr..

[47] Soldavini J., Berner M. (2020). The importance of precision: Differences in characteristics associated with levels of food security among college students. Public Health Nutr..

[48] NutritionQuest Web-Based Wellness Solutions. Free Assessment Tools for Individuals. https://nutritionquest.com/wellness/free-assessment-tools-for-individuals/.

[49] (2015). Laerd Statistics. Ordinal Logistic Regression Using SPSS Statistics. Statistical Tutorials and Software Guides. https://statistics.laerd.com/.

[50] Lopez A. (2023). “Money for Food”: A Deeper Look at Food Insecurity and Class Privilege in Higher Education.

[51] Dennard E., Kristjansson E., Tchangalova N., Totton S., Winham D., O’connor A. (2022). Food insecurity among African Americans in the United States: A scoping review. PLoS ONE.

[52] Hamilton M., Morrow B.F., Davis L.B., Morgan S., Ivy J.S., Jiang S., Chi M., Hilliard K. (2024). EXPRESS: Toward a More Diverse and Equitable Food Distribution System: Amplifying Diversity, Equity and Inclusion in Food Bank Operations. Prod. Oper. Manag..

[53] Brown N.I., Buro A.W., Jones R., Himmelgreen D., Dumford A.D., Conner K., Stern M., DeBate R. (2023). Multi-Level Determinants of Food Insecurity among Racially and Ethnically Diverse College Students. Nutrients.

[54] Winham D.M., Florian T.L.A. (2015). Nativity, not acculturation, predicts SNAP usage among low-income Hispanics with food insecurity. J. Hunger. Environ. Nutr..

[55] El Zein A., Shelnutt K.P., Colby S., Vilaro M.J., Zhou W., Greene G., Olfert M.D., Riggsbee K., Morrell J.S., Mathews A.E. (2019). Prevalence and correlates of food insecurity among U.S. college students: A multi-institutional study. BMC Public Health.

[56] Williams D.R. (2002). Racial/ethnic variations in women’s health: The social embeddedness of health. Am. J. Public Heal.

[57] Wright K.E., Lucero J.E., Ferguson J.K., Granner M.L., Devereux P.G., Pearson J.L., Crosbie E. (2021). The influence of cultural food security on cultural identity and well-being: A qualitative comparison between second-generation American and international students in the United States. Ecol. Food Nutr..

[58] Winham D.M. (2009). Culturally Tailored Foods and Cardiovascular Disease Prevention. Am. J. Lifestyle Med..

[59] Cummings J., Bergquist E., Boateng L., Phoxay C., Stadler D. (2021). Innovative Partnerships: Education that bridges cultures to develop globally fluent dietitians and nutritionists. Top. Clin. Nutr..

