# Supplementary material for: Food Insecurity Predictors Differ for White, Multicultural, and International College Students in the United States
Source: Nutrients. 2025 Jan 10;17(2):237. doi: 10.3390/nu17020237 (PMC11767435; doi:10.3390/nu17020237)
Supplement: Supplementary file 1 [file nutrients-17-00237-s001.zip › nutrients-3397186-supplementary.pdf]

Supplemental Table S1. Food security category classification and individual module responses for the past 6 months by nativity–ethnicity among Midwest university students (n=853) [3,38,40]

	Total	US White (37%; 316)	Multicultural (28%; 239)	International (35%; 298)	p
Food Security Screening Questions		←	%	→	
In last 6 months I/we had…					n.s.
Enough of kinds of food I want to eat	49.2	54.7	44.4	47.3	
Enough, not always kinds of food I want	44.4	39.9	48.1	46.3	
Sometimes not enough to eat	5.0	5.1	5.9	4.4	
Often not enough to eat	1.3	0.3	1.7	2.0	
Of the 49.2% (420/853) who said had "enough of kinds of food to eat" …		US White (41%;173)	Multicultural (25%; 106)	International (34%; 141)	
Run out of food at the end of the month (% of time)					0.026
Never (subsequently classified as food secure)	76.7	83.8_a	68.9_b	73.9_b	
Seldom (25%)	16.2	9.8_a	24.5_b	17.6_b	
Sometimes (50%)	3.8	3.5_a	1.9_a	5.6_a	
Most times – Always (75-100%)	3.3	2.9_a	4.7_a	2.8_a	
Food Security Module Questions: Of the 62.1% (530/853) who may be food insecure:	Total	US White (32%;172)	Multicultural (31%; 174)	International (36%; 204)	
Food bought did not last, no have money to get more					0.207
Often true-Sometimes true	51.5	48.0	59.0	48.2	
Never true	48.5	52.0	41.0	51.8	
Could not afford to eat balanced meals					0.399
Often true-Sometimes true	57.9	54.4	63.9	56.0	
Never true	42.1	45.6	36.1	44.0	
Worried food would run out before got money					0.011
Often true-Sometimes true	41.3	34.5	44.6	44.6	
Never true	58.7	65.5	55.4	55.4	
Cut size or skipped meals because not enough money					0.614
Yes	26.0	22.8	28.9	26.4	
No	74.0	77.2	71.1	73.6	
Did not eat for a whole day because there was not enough money for food.					0.355
Yes	8.7	5.9	10.2	9.9	
No	91.3	94.1	89.8	90.1	
Ate less than felt should because not enough money					0.182
Yes	31.4	31.2	33.1	30.1	
No	68.6	68.8	66.9	69.9	
Was hungry but did not eat, no money for food.					0.127
Yes	22.7	22.9	25.3	20.3	
No	77.3	77.1	74.7	79.7	
Lost weight because not enough money for food.					0.375
Yes	10.8	9.4	13.3	9.8	
No	89.2	90.6	86.7	90.2	

Same subscript letters indicate column proportions that are not significantly different from each other; n.s.=not significant. Values of $p < 0.05$ are considered statistically significant.

Supplemental Table S2. Mean consumption frequency[1] of individual food items from the dietary fat food frequency screener nativity–ethnicity groups of Midwest university students (%; *n* = 853) [36]

FAT SCREENER FOOD ITEMS	Total	US White (37%; 316)	Multicultural (28%; 239)	International (35%; 298)	*p*
Eggs (not Egg Beaters or egg whites)	3.12 ± 1.3	2.89 ± 1.2$_a$	3.00 ± 1.3$_a$	3.46 ± 1.31$_b$	<0.001
Cheese or cheese spreads (not low-fat)	3.00 ± 1.3	3.37 ± 1.2$_a$	2.99 ± 1.3$_b$	2.61 ± 1.3$_c$	<0.001
Corn chips, potato chips, popcorn, crackers	2.77 ± 1.1	2.96 ± 1.1$_a$	2.90 ± 1.1$_a$	2.47 ± 1.1$_b$	<0.001
Margarine, butter, or mayonnaise on foods	2.68 ± 1.2	2.84 ± 1.2$_a$	2.71 ± 1.1$_a$	2.50 ± 1.2$_b$	0.002
French fries, fried potatoes	2.51 ± 1.0	2.56 ± 1.0$_a$	2.71 ± 1.0$_a$	2.29 ± 1.0$_b$	<0.001
Hamburgers, cheeseburgers, ground beef	2.50 ± 1.1	2.77 ± 1.1$_a$	2.68 ± 1.1$_a$	2.07 ± 1.1$_b$	<0.001
Doughnuts, pastries, cake, cookies	2.44 ± 1.1	2.49 ± 1.1$_a$	2.54 ± 1.1$_a$	2.31 ± 1.1$_b$	0.027
Whole milk (not low-fat or skim)	2.34 ± 1.5	2.04 ± 1.3$_a$	2.09 ± 1.4$_a$	2.84 ± 1.6$_b$	<0.001
Pizza	2.21 ± 0.9	2.35 ± 0.8$_a$	2.27 ± 1.0$_a$	2.02 ± 0.9$_b$	<0.001
Cold cuts, lunch meats, ham	2.14 ± 1.2	2.41 ± 1.2$_a$	2.21 ± 1.1$_b$	1.80 ± 1.1$_c$	<0.001
Salad dressings (not low-fat)	2.10 ± 1.1	2.29 ± 1.1$_a$	1.94 ± 1.0$_b$	2.01 ± 1.0$_b$	<0.001
Bacon or breakfast sausage	1.83 ± 1.0	1.94 ± 1.0$_a$	1.93 ± 1.0$_a$	1.62 ± 1.0$_b$	<0.001
Fried chicken	1.81 ± 1.0	1.60 ± 0.8$_a$	1.73 ± 0.9$_a$	2.10 ± 1.1$_b$	<0.001
Not significant by Nativity-Ethnicity:					
Margarine, butter, or oil in cooking	3.64 ± 1.2	3.61 ± 1.1	3.64 ± 1.2	3.66 ± 1.4	0.892
Beef, pork, e.g., steaks, roast, ribs, or sandwiches	2.38 ± 1.2	2.40 ± 1.1	2.41 ± 1.1	2.34 ± 1.3	0.735
Ice cream (not sherbet or low-fat)	2.11 ± 1.0	2.14 ± 1.0	2.12 ± 1.0	2.08 ± 1.0	0.781
Hot dogs, or Polish or Italian sausage	1.49 ± 0.8	1.53 ± 0.7	1.49 ± 0.8	1.45 ± 0.7	0.379

[1] Food frequency response options: 1 = 1 time a month or less; 2 = 2-3 times a month; 3 = 1-2 times a week; 4 = 3=4 times a week; 5 = 5 times or more a week [39]. Same subscript letters (a, b) indicate column proportions that are not significantly different from each other.

Supplemental Table S3. Requested food items from on-campus food pantry users by nativity–ethnicity from Midwest uni-versity students (n = 51)

Requested Items	Total (n = 51)	US White (9.8%; 5)	US Multicultural (27.5%; 14)	International (62.7%; 32)
**Food Categories**				
Produce	24	-	6	18
Fruits	27	3	9	15
Vegetables	16	-	3	13
Grain	18	1	5	12
Meat/Protein	12	1	4	7
Eggs	12	1	3	8
Pulse/Legume	8	-	1	7
Plant-based Meat Alternative	1	-	1	-
Dairy	11	1	2	8
Non-dairy Milk	3	-	1	2
Protein Bar	1	-	-	1
Nuts	1	-	-	1
Juice	3	1	1	1
Fats	2	-	-	2
Baby food	1	-	-	1
Chips	1	-	-	1
Seasonings/spices/sauces	8	-	3	5
Desserts	1	-	-	1
**Food Themes**				
Fresh/perishable	10	-	4	6
More variety	5	1	4	-
Easy to prepare	4	-	1	3
Basics	2	-	1	1
Full meal	5	1	2	2
**Dietary Preferences**				
Cultural foods	4	-	1	3
Religion	2	-	-	2
Reference to allergy/intolerance	2	-	-	2
Vegetarian diet	2	1	-	1
**Non-food Items**				
Household cleaning/toiletries	3	1	-	2
